# Sequence-Based Indoor Localization with Channel Status Information

**DOI:** 10.3390/s18061818

**Published:** 2018-06-04

**Authors:** Zhongqiu Wang, Ying Chen, Hai Wang

**Affiliations:** School of Computer Science and Technology, China University of Mining and Technology, Xuzhou 221116, China; wzq-135@163.com (Z.W.); wh_cumt@cumt.edu.cn (H.W.)

**Keywords:** received signal strength indication, indoor localization, access point, channel status information

## Abstract

Most of the indoor localization systems nowadays are based on received signal strength indication (RSSI), which has further increased the importance of precise localization of access points (AP) in a wireless local area network (WLAN). Since most existing AP localization algorithms suffer from a high error rate in practical scenarios due to multipath fading and temporal dynamics, we propose an AP localization algorithm based on the channel status information (CSI) sequence-based localization (SBL-CSI). The proposed algorithm SBL-CSI is an efficient localization method that consists of the following three major steps: Firstly, a 2D localization space is divided by special APs into distinct regions, and each region has a unique location sequence that represents the distance ranks of special APs to that region and constructs the location sequence table. Then, the relative distance of the ordinary AP, served in the location sequence, is obtained by using CSI between the ordinary AP and special AP. Finally, the “nearest” feasible sequence of the ordinary AP in the location sequence table is searched, and the centroid of the corresponding region is the ordinary AP’s localization. Compared with the traditional localization algorithm based on RSSI, the experiment results demonstrate that the positioning accuracy is improved approximately 24.31%.

## 1. Introduction

Wireless indoor positioning generates many applications based on geographical location in a wide range of life, production, business and public services. While the Global Positioning System (GPS) performs well in outdoor localization, it does not work effectively in indoor circumstances. A well-known bottleneck of the GPS indoor environment is that the transmission of the satellite signal [1] will be blocked by buildings, and the positioning accuracy of GPS will be greatly limited. Therefore, indoor localization is more challenging and has been gaining growing attention with the flourishing of mobile and pervasive computing. Pioneering works in this field can be roughly categorized as either requiring measurement of the the distance or angle of nodes or no measurement in the positioning process.

Due to the popularization of intelligent terminal equipment, indoor localization service [2] is one of most challenging problems in wireless communication technology. There is a series of ground wireless positioning systems based on localization technology, such as ultrasonic [3], Bluetooth [4,5] and ultra-wideband (UWB) [6,7]. Localization technology based on WLAN positioning technology [8,9] has become a research hotspot, because it has the advantages of convenient access, low cost, wide coverage and fast communication. The existing WLAN topology can be divided into two types: a self-organizing network and an infrastructure network. An infrastructure network is widely used where the AP of WLAN is in charge of the coverage and communication of the wireless network.

Despite the increasing sophistication of indoor localization systems, precise indoor localization is still a critical problem. In this paper, we research the localization of wireless access points, the significance of which is that with the development of high-density WLAN, the coordination and management of wireless networks has become a critical issue and determines the APs’ location by using their signals. For individual users, AP localization can help them access the AP’s location information and quickly connect to the network. For network administrators, AP localization makes a great contribution to network management, identifies additional APs and finds illegal APs [10]. Especially in some classified units, AP localization can be found to locate other APs. The key contributions of our work are broken down into the following aspects:

(i) We exploit the CSI location sequence to stretch the limit towards RSSI value localization accuracy.
(ii) We develop a sequence-based localization (SBL)-CSI localization framework, which leverages fine-gained PHY layer information to improve AP positioning performance.
(iii) We conduct extensive experiments to show the feasibility of our design. The performance of the proposed design is evaluated by data collected in the experiment. The results show that SBL-CSI achieves better localization accuracy compared to SBL-RSSI.

The remainder of this paper is structured as follows: the related works are briefly presented in Section 2. Section 3 presents the SBL-CSI algorithm design. The system design is described in Section 4, followed by a comprehensive experimental evaluation in Section 5. Finally, Section 6 concludes the article.

## 2. Related Work

Wireless localization in WLAN has attracted extensive research attention. Currently, there exist two types of wireless localization methods: range-based localization [11,12,13,14,15,16,17] and range-free localization [18,19,20], according to whether there is actually the distance or angle of measured nodes in the localization process. The range-based localization mechanisms determine the localization of unknown nodes by measuring the actual point-to-point distance or angle information between nodes and using the trilateration, triangulation or maximum likelihood estimation methods, based on the time of arrival (TOA) [11], time difference of arrival (TDOA) [12,13], angle of arrival (AOA) [14], signal-based signature distance [15], received signal strength indicator (RSSI) [16,17], and so on. In the above four algorithms, TOA, TDOA and AOA require additional hardware devices, while RSSI does not because the interface of most nodes has the function of receiving radio frequency (RF) signals [21] and can test the signal strength. The key of RSSI-based localization is to obtain the RF signal strength, which introduces extra hardware cost and deployment complexity. However, the disadvantage of the RSSI localization algorithm is that the propagation of the wireless signal is seriously affected by the environment. Range-free localization does not measure the distance between nodes; the coordinate of the node can be calculated according to the connectivity of the network and the information of the beacon nodes, such as the DV-Hop algorithm [18,19] and APIT [20]. Compared with range-based localization, range-free localization does not require complicated hardware support; nevertheless, it has lower positioning accuracy.

Sequence-based localization (SBL) [22] is an integrated algorithm based on range-based localization and range-free localization used in recent years. The core idea of this scheme is to leverage a two-dimensional (2D) plane where ranked nodes are spatially divided in some way to construct the virtual beacon nodes. Then, the order of the virtual beacon nodes is determined based on the distance order of the virtual beacon nodes, that is the location sequence.

The existing methods for positioning APs are mostly RSSI localization techniques. The work in [23] is based on the gradient values of RSSI, measuring at multiple points, comparing the received signal strength values to analyze the trend of RSSI and estimating the location of the AP. To further improve the positioning accuracy, Zhao et al. proposed an RSSI gradient-based AP localization algorithm [24]. In this algorithm, the node estimates the direction of the AP based on the received local signal strength, selects and filters the outliers using the clustering method and finally calculates the coordinate of the wireless AP using the triangulation method. By analyzing the statistical characteristics of the RSSI difference in heterogeneous devices, a normal distributed model is introduced by Young et al., which is used to correct RSSI measurement to improve the accuracy of AP position [25]. To construct a relative map of all WiFi AP positions [26], the dissimilarities between pairs of APs can generate a geometric configuration of APs based on the multidimensional scaling technique. Experimental results show WiFi AP positioning errors within 10 m. The work in [27], based on signal propagation model, obtained a set of RSSI measured at one or more points and then used the wireless signal propagation model to convert signal strength into the distance between the measuring point and the AP and, finally, calculated the location of the AP. An indoor localization system employed an ordered sequence of APs based on RSS [28], and it avoided any time-consuming calculations to characterize radio signals in the environment.

In order to improve the positioning accuracy, the state-of-the-art wireless localization schemes collect more data at many points and angles, which requires time and effort. This paper demonstrates that the sequence localization algorithm in [22] can indeed be exploited for high accuracy, and we propose a WLAN access point localization algorithm based on the CSI sequence to position the wireless AP. Our work achieves higher precision positioning by dividing the whole experimental area and establishing a corresponding location sequence table, where the value of each location sequence is the distance between the centroid of the divided area and the known location of the AP. When measuring the located AP, the “nearest” sequence of the location table is searched by comparing the location sequence of the located AP according to CSI, and the centroid of the representative area is the estimated position of the located AP. Given the number of APs with known location, we optimize the correlation coefficient between the two sequences so that it can further boost the localization accuracy. The experimental results show that the average localization error of the SBL-CSI algorithm is far lower than that of the algorithm proposed by [22].

## 3. Overview of the SBL Localization Algorithm Design

In this section, we introduce the application of the sequence location algorithm to position APs in WLAN. We first define the WLAN network model, followed by introducing the SBL localization algorithm.

### 3.1. Network Model

With the wide coverage of wireless APs in the indoor environment, users can access the Internet at anytime and anywhere via a portable wireless communication device. Sometimes, we need to know the location of APs, whose location is unknown. According to the ability to parse CSI, we divide APs into two categories: special AP and normal AP. Normal APs are commercially available wireless APs, but do not have the ability to parse CSI, and their location is unknown. Compared with normal APs, special APs are equipped with special configurations, such as having the configuration of the wireless network card and operating system to parse CSI, and their location can be known by GPS or other methods. Considered the cost of a special AP and the necessity of the location of all APs, there are only a few special APs’ locations that are known when deploying APs. To know the location information of some normal APs, we estimate the location of normal APs based on the special APs.

**Figure 1:**
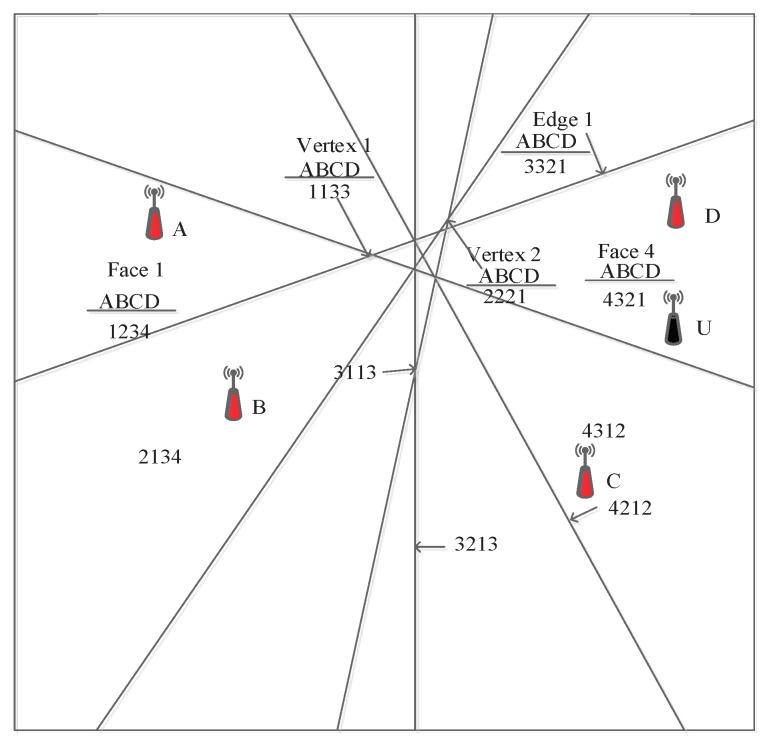
Schematic diagram of sequence-based localization (SBL).

We assume that *N* special APs are distributed in 2D localization space, where a perpendicular bisector is drawn between any two APs; there should be $\frac{N(N-1)}{2}$ perpendicular bisectors. As shown in Figure 1, A, B, C and D represent special APs and U stands for the ordinary AP. The perpendicular bisector separates 2D space into three types: vertices, edges and faces, calculating the centroid of them via Equations (1)–(4). Then calculating the Euclidean distance *d* between a centroid and special APs in the localization space, we can obtain the ordered sequence for that centroid with respect to the location of the special APs. We define the ordered sequence as the location sequence. Based on the above division, the location sequence of a given region is unique to that region [22], which is used for positioning normal APs.

### 3.2. SBL Localization Algorithm

The process of using the location sequences to position a normal AP is as follows:

1. Determine the location sequence of all divided regions and list them in the form of a location sequence table.
2. Establish the location sequence of the ordinary APs by using the RSSI measurement method, which determines the distance from the ordinary APs to each special AP.
3. According to Equation (5), calculate the maximum Kendall’s Tau between the location sequence of ordinary APs and other sequences in the location sequence table. The centroid mapped to that sequence is the location estimate of ordinary APs.

The location sequence of normal APs is determined by a predefined order of special AP IDs. We illustrate the above procedures of generating location sequences through Figure 1. There are four special APs; the number of perpendicular bisectors is six; those divide the localization space into multiple regions. The predefined sequence of special AP IDs is ABCD. For the four special APs, the closest distance to the centroid of Face 1 is A, followed by B, C and D; therefore, the location sequence of Face 1 is defined as 1234. In contrast, for Face 4, the location sequence is 4321, because the distance rank of A is the farthest, while D is the closest, and C is closer than B and A. Similarly, the location sequence of *U* is 4321. The centroid of Edge 1 is exactly on the perpendicular bisector of two APs A and B; consequently, the distance of two APs is equal. C is closer to that edge than APs A and B, and the nearest AP is D. Therefore, the location sequence is 3321. Vertex 1 is on the perpendicular bisectors of two pairs A, B and C, D, respectively, but closer to the A, B pair; thus, the location sequence in this region is 1133. The location sequence of Vertex 2 is 2221, as D is the nearest (distance Rank 1), and the distance ranks of A, B and C are the same, due to lying on the perpendicular bisectors of the A, C and B, C pairs.

A few related concepts in the sequence localization algorithm are further explained below.

• Get Edge Centroid.

Suppose that ($C_{x}$, $C_{y}$) is the centroid of an edge *C*, whose midpoint is given by:

(1) $$\left(C_{x},C_{y}\right)=\left(\frac{O_{x}+D_{x}}{2},\frac{O_{y}+D_{y}}{2}\right)$$

where ($O_{x}$, $O_{y}$) and ($D_{x}$, $D_{y}$) are the starting and ending points of the edge, respectively.

• Get the face centroid:

The centroid of a face is the geometric center of the polygon; ($P_{x}$, $P_{y}$) is the centroid coordinate of the face *P*, given its vertices $\left\{(x_{i},y_{i})|0\leq i\leq p-1\right\}$, calculated by:

(2) $$\begin{array}{c} P_{x}=\frac{1}{6A}\sum_{i=0}^{p-1}\left(x_{i}+x_{i+1}\right)\left(x_{i}y_{i+1}-x_{i+1}y_{i}\right) \end{array}$$

(3) $$\begin{array}{c} P_{y}=\frac{1}{6A}\sum_{i=0}^{p-1}\left(y_{i}+y_{i+1}\right)\left(x_{i}y_{i+1}-x_{i+1}y_{i}\right) \end{array}$$

where *A* is the area given by:

(4) $$\begin{array}{c} A=\frac{1}{2}\sum_{i=0}^{p-1}\left(x_{i}y_{i+1}-x_{i+1}y_{i}\right) \end{array}$$

• Distance measurement index:

The order of the location sequences is determined by the distance between positioned APs and special APs; the statistics declares a metric that presents the difference in the two rank orders. That is Kendall’s Tau τ, which is used to reflect the difference arrangement order of two location sequences as a measurement of distance. Given two location sequences $U=\left\{u_{i}\right\}$ and $V=\left\{v_{i}\right\}$, 1≤i≤N, where $u_{i}$ and $v_{i}$ are the sequence of special APs, the τ coefficient elaborates the correlation between the relative order of the two sequences. It compares all $\frac{N(N\;-\;1)}{2}$ pairs of ranks $\left(u_{i},v_{i}\right)$ and $\left(u_{j},v_{j}\right)$ to determine how many matching pairs and non-matching pairs there are. A pair is matching or has a correlation if $u_{i}>u_{j}\Rightarrow v_{i}>v_{j}$ or $u_{i}<u_{j}\Rightarrow v_{i}<v_{j}$ and is non-matching if $u_{i}>u_{j}\Rightarrow v_{i}<v_{j}$ or $u_{i}<u_{j}\Rightarrow v_{i}>v_{j}$. Kendall’s Tau τ is given by [22] :

(5) $$\begin{array}{c} \tau =\frac{n_{c}-n_{d}}{\sqrt{n_{c}+n_{d}+n_{tu}}\sqrt{n_{c}+n_{d}+n_{tv}}} \end{array}$$

where $n_{c}$ is the number of matching pairs, $n_{d}$ is the number of non-matching pairs, $n_{tu}$ is a number of equal value in sequence *U* and $n_{tv}$ is a number of equal value in sequence *V*. The range of Kendall’s Tau τ is [−1,1].

The primary advantage of the SBL algorithm is that it accomplishes localization work with low requirements for hardware, whereas it needs the collection of the location sequence table information and to centralize it to compute the data. However, the SBL algorithm only focuses on RSSI, which still suffers from coarse granularity and high susceptibility to the multipath fading effect in indoor environments. As a result, the value of RSSI propagates over distance and attenuates, but not following an inverse-square law, which incurs low ranging precision and localization accuracy. We propose a WLAN access point location optimization scheme based on the SBL algorithm.

## 4. System Design

We analyze the insufficiency of the literature SBL algorithm, and then, we introduce our detailed design for improving accurate localization in wireless sensor network applications.

### 4.1. Deficiencies in the SBL Algorithm

The sequence-based localization algorithm has a good theoretical basis and effective positioning; however, there are still the following problems:

1. The SBL algorithm is the solution that calculates the location sequences of normal APs based on RSSI, while RSSI suffers from dramatic performance degradation in complex situations due to multipath fading and temporal dynamics.
2. For the distance measurement index in SBL, Kendall’s Tau τ is a measurement of the proximity degree for two sequences, the length of which is related to the number of special APs, and the correlation of the two sequences increases as the number of special APs increases. We consider the role of the number of special APs in the order of sequences to improve the Kendall coefficient.

This foregoing analyses are applied without considering the existence of obstructions like walls, devices and moving humans. If obstructions need to be considered, we will further modify the algorithm from the two following aspects.

1. Different from RSSI as the MAC layer is susceptible to multipath characteristics with fast changes, the power feature of the PHY layer, CSI has the capacity to attenuate the multipath fading effect. Based on the CSI measurement method, this paper finds the relative distance of normal APs from each special APs, then obtains the location sequence of normal APs and afterwards uses the SBL algorithm to find out the estimated coordinate of ordinary APs.
2. In order to optimize the correlation between two sequences, we make the following change to Equation (5) [29].
  (6) $$\begin{array}{c} \tau^{\prime }=\frac{n_{c}^{\prime }-n_{d}^{\prime }}{\sqrt{n_{c}^{\prime }+n_{d}^{\prime }+n_{tu}^{\prime }}\sqrt{n_{c}^{\prime }+n_{d}^{\prime }+n_{tv}^{\prime }}} \end{array}$$
  where, if $u_{i}>u_{j}\Rightarrow v_{i}>v_{j}$ or $u_{i}<u_{j}\Rightarrow v_{i}<v_{j}$, then $n_{c}^{\prime }=n_{c}^{\prime }+\lambda \left(1-u_{i}/\lambda \right)$; if $u_{i}>u_{j}\Rightarrow v_{i}<v_{j}$ or $u_{i}<u_{j}\Rightarrow v_{i}>v_{j}$, then $n_{d}^{\prime }=n_{d}^{\prime }+\lambda \left(1-u_{i}/\lambda \right)$; if $u_{i}=u_{j}$, then $n_{tu}^{\prime }=n_{tu}^{\prime }+\lambda \left(1-u_{i}/\lambda \right)$; if $v_{i}=v_{j}$, then $n_{tv}^{\prime }=n_{tv}^{\prime }+1$, and λ is the number of special APs.

### 4.2. CSI Measurement Method and Distance Estimation

We illustrate a better way to improve the accurate indoor localization to find another counterpart of RSSI that is more stable and holds potential for the convergence of accurate and pervasive indoor localization.

### 4.3. Stability Test of CSI

For WiFi signal utilizing the 802.11n transmission protocol [30], which uses orthogonal frequency division multiplexing (OFDM) modulation to transmit the signal over multiple orthogonal subcarriers, the signal transmitted on each subcarrier has a different signal strength and phase. Specifically, using an off-the-shelf Intel 5300AGN NIC and a modified set of drivers, a sample version of the channel frequency response (CFR) in the WiFi bandwidth can be output in the form of CSI. Each of CSI value represents the amplitude and phase information of one subcarrier in the transmit-receive antenna [31].

(7) $$H\left(f\right)=∥H\left(f_{i}\right)∥e^{j\;\sin\{∠H\left(f_{i}\right)\}}$$

where $H(f_{i})$ is the CSI at the subcarrier with the frequency of $f_{i}$, $∥H\left(f_{i}\right)∥$ represents its amplitude and $∠H(f_{i})$ is the phase.

$H(f_{i})$ describes the amplitude and phase information of the subcarrier group $f_{i}$, and the channel impulse response (CIR) can be obtained by utilizing the inverse fast Fourier transform (IFFT) of CFR:

(8) h(τ)=IFFT(H(f))

**Figure 2:**
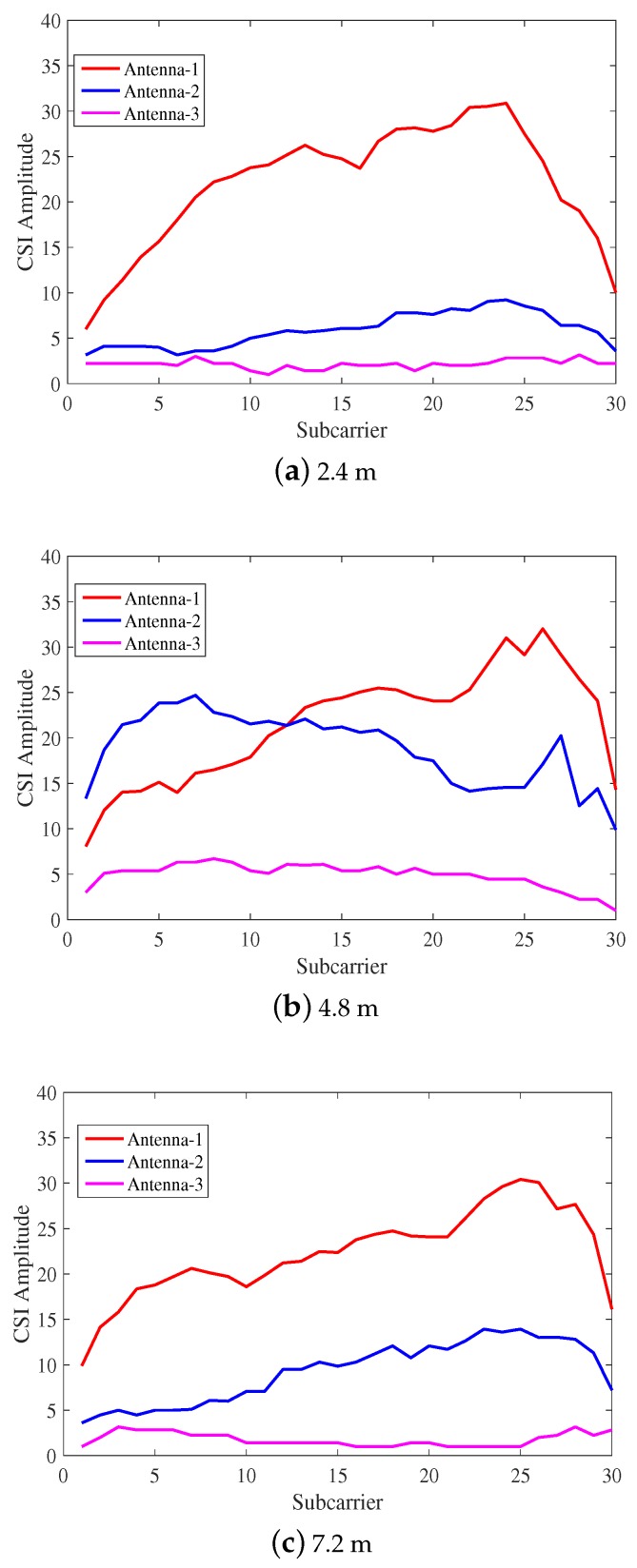
The channel status information (CSI) amplitude changes under different distances of the indoor open environment.

Figure 2 depicts the variation of CSI amplitude in the indoor open environment. Figure 2a–c shows the CSI amplitudes of the received signals corresponding to 2.4 m, 4.8 m and 7.2 m from the AP, respectively. It can be seen that with the increase of distance, the CSI amplitude of the received signal of the same antenna decreases. The reason for this situation is that the value of CSI propagates over distance and attenuates following an inverse-square law, the same as RSSI.

**Figure 3:**
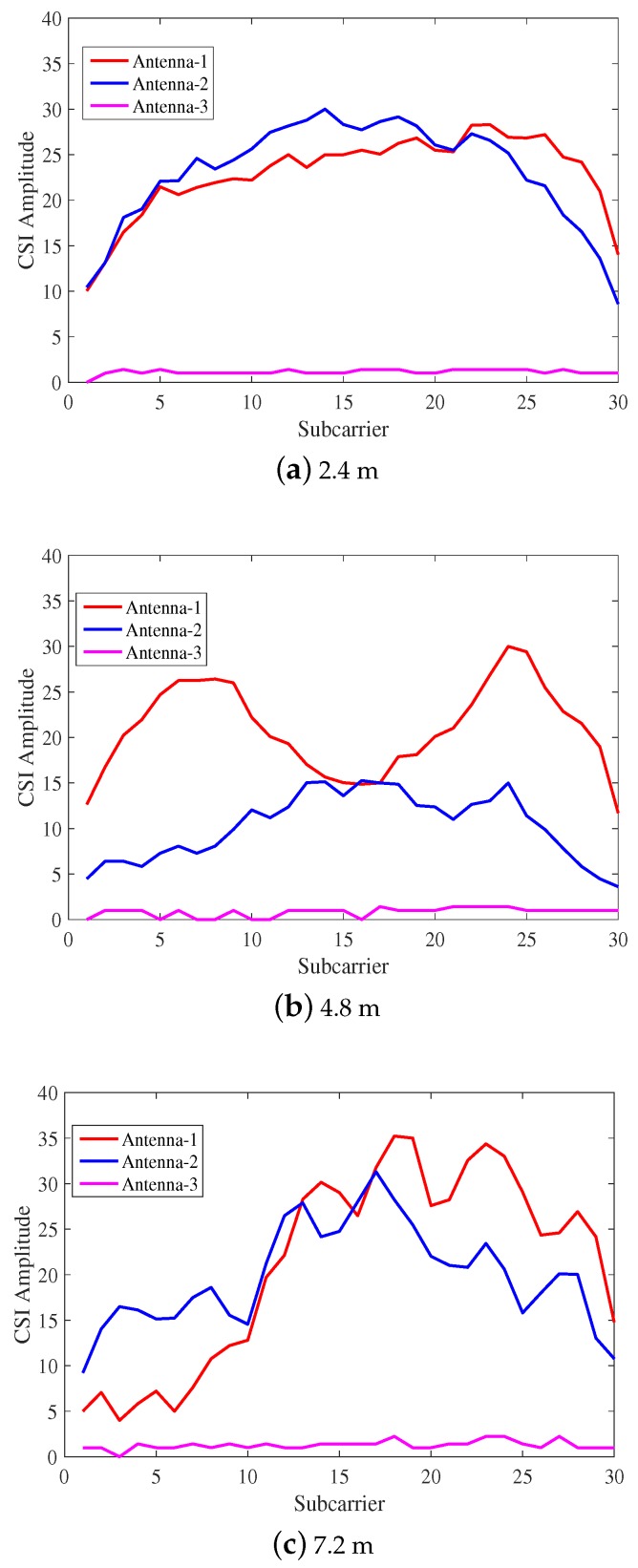
The CSI amplitude changes under different distances of indoor complex environment.

Figure 3 shows that the CSI amplitude from the receiver in the indoor complex environment. By comparison, we discover that the change of CSI amplitude of different subcarriers collected in the open environment is relatively stable, and that collected in the complex environment has the most dramatic changes. This is because, in the indoor complex environment, due to the presence of plants, walls, tables and other objects, the wireless signal in the propagating process is no longer simply the line-of-sight [32], producing reflection, diffraction and scattering phenomena. In the complex environment, wireless signal propagation is much more complicated than in the free space environment.

**Figure 4:**
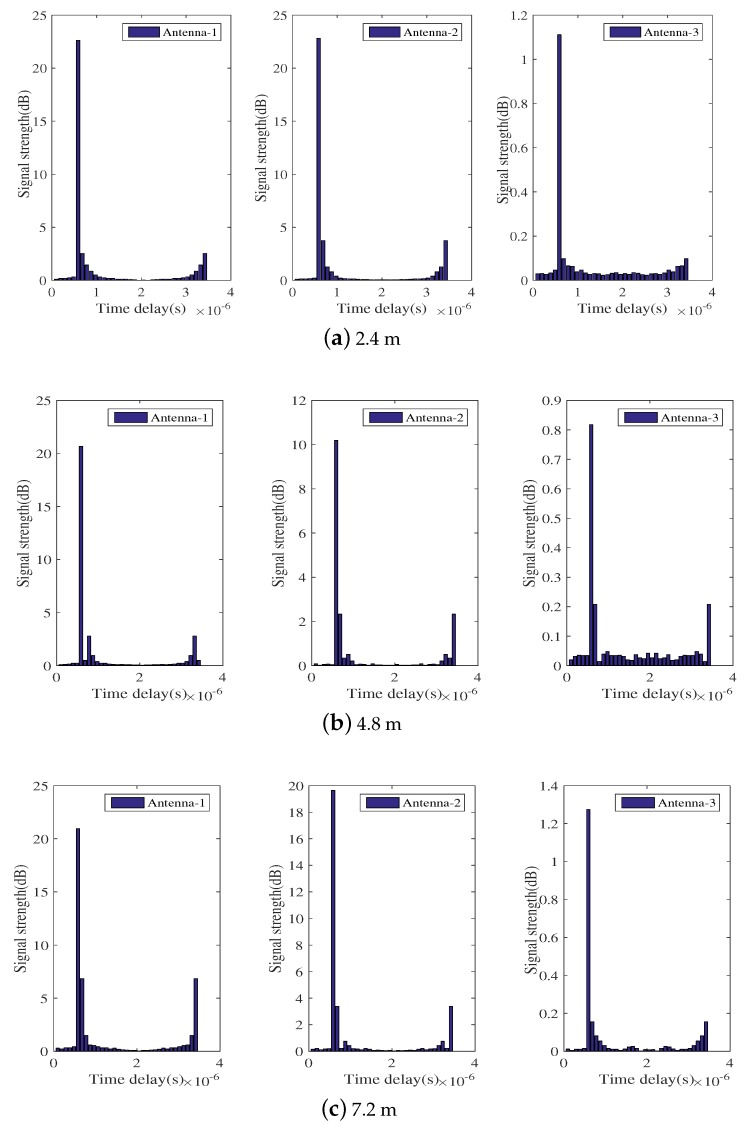
Time domain diagram of the indoor laboratory at different distances.

By applying IFFT to the frequency domain data, we plot the CIR diagram of the time domain, where CIR characterizes the relationship between time delay and signal strength. As shown in Figure 4, the time interval between two adjacent straight bars is 50 ns, and the ordinate indicates the signal energy received at the receiver under the corresponding delay. Figure 4 shows the CIR at various distances in the laboratory with obstacles, and the three plots have very small energy values at the lowest delay.

In theory, the line-of-sight (LOS) path propagates a shorter distance than the non-line-of-sight (NLOS) path and preferentially arrives at the receiver. Therefore, in the ideal case, the first bar in the CIR plot represents the energy of the LOS path. However, in fact, there is an uncertain time delay due to phase drift or other factors at the shortest time delay. This is effective data after the uncertain time delay. Therefore, there exists a situation where LOS path and NLOS path energy are superimposed on the same bar in the CIR graph, as shown in Figure 4. Thus, simply calculate the energy value corresponding to the first bar of the CIR graph or the bar of the first maximum value, as the LOS path energy has a relatively large error. To calculate effective CSI data, the original data should have noise reduction processing and filtering performed for the influence of the time delay.

### 4.4. CSI Data Noise Reduction

**Figure 5:**
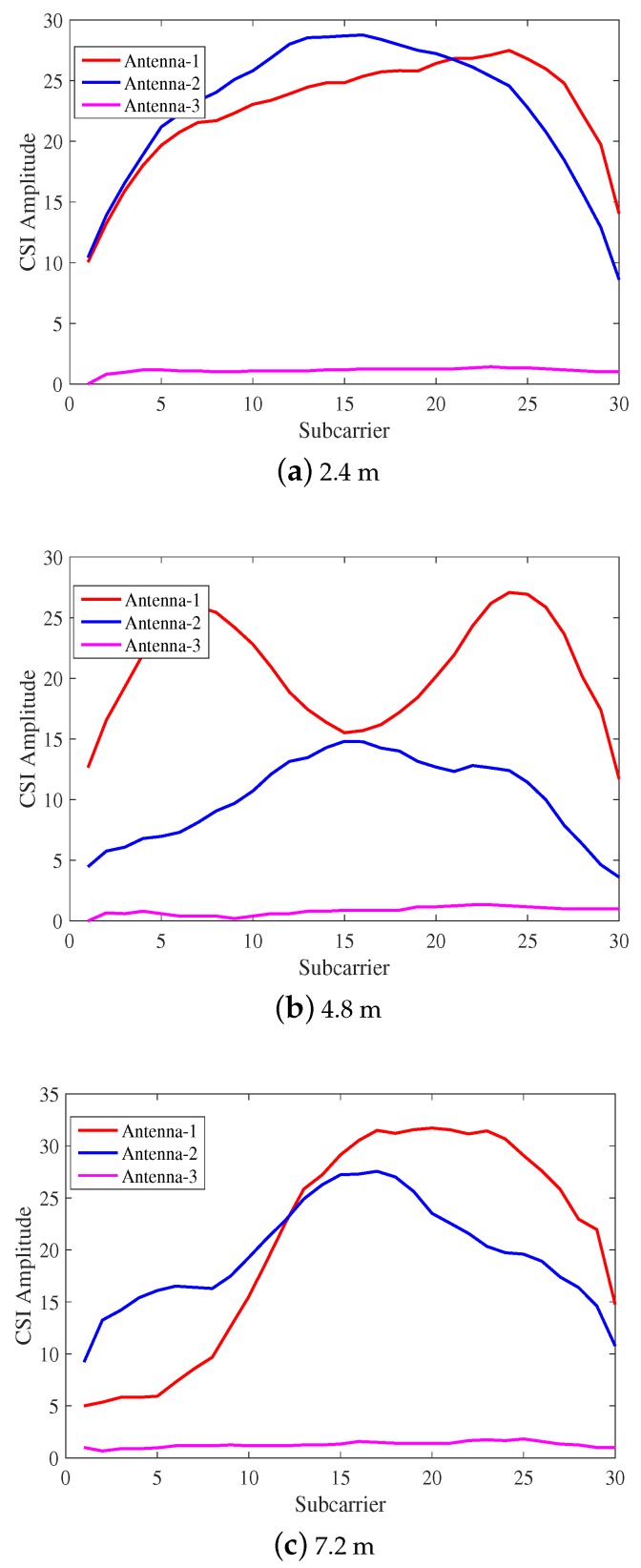
The CSI data noise reduction under different distances of indoor complex environments.

The noise signal has a significant impact on multipath elimination, which leads to the reduction of the positioning accuracy. However, since the interference signal consists of random noise and high frequency noise, it is unreasonable to simply consider the interference signal as random noise. If collected CSI values in complex environments are directly substituted into the ranging model, there will be a very large location error. Therefore, we eliminate the multipath fading effect before establishing the mapping relationship between CSI and distance. In order to calculate the effective CSI data, we first reduce the influence of random noise by linearly filtering the original frequency domain data and then use the Butterworth filter to filter out the environmental changes and other factors that lead to high frequency noise. Figure 5 shows that the denoising of CSI data under different distances of complex environments.

• Linear filtering:

Due to the influence of random noise, the CSI phase information is rarely used for indoor positioning systems. In this paper, the obtained original CSI data are linearly transformed to mitigate the effect of random noise on the phase information. Assuming that $\hat{\psi }$ represents the phase measurement of subcarrier *i*, then $\hat{\psi }$ can be expressed as:

(9) $$\hat{\psi }=\psi -2\pi \frac{K_{i}}{N}\Delta t+\varepsilon +O$$

where ψ is the original phase information, Δt is the timing offset at the receiver, an important factor that causes the phase error, ε is unknown phase offset, *O* is the noise, $k_{i}$ is the index of the *i*-th subcarrier and *N* is the sample number of the fast Fourier transform (FFT).

The core idea of linear filtering is to eliminate the timing offset Δt and the phase offset ε by using the phase of the entire frequency band. We define two parameters *p* and *q*:

(10) $$p=\frac{{\hat{\psi }}_{n}-\psi_{1}}{k_{n}-k_{1}}=\frac{\psi_{n}-\psi_{1}}{k_{n}-k_{1}}-\frac{2\pi }{N}\Delta t$$

(11) $$\begin{array}{c} q=\frac{1}{n}\sum_{j=1}^{n}{\hat{\psi }}_{j}=\frac{1}{n}\sum_{j=1}^{n}\psi_{j}-\frac{2\pi \Delta t}{nN}\sum_{j=1}^{n}k_{j}+\varepsilon \end{array}$$

Ignoring the smaller measurement noise *O*, we can get a pure phase linear combination $\tilde{\psi }$, given by:

(12) $$\tilde{\psi }=\hat{\psi }-pk_{i}-q=\psi_{i}-\frac{\psi_{n}-\psi_{1}}{k_{n}-k_{1}}k_{i}-\frac{1}{n}\sum_{j=1}^{n}\psi_{j}$$

• Butterworth filtering:

The original data are inevitably mixed with a certain degree of high frequency noise because of environmental factors. We use the Butterworth filter to eliminate the effect of high-frequency noise on the amplitude information. The gain of the *N*-order Butterworth filter is generally expressed by Equation (13), where ε is a constant whose value should satisfy $\varepsilon^{2}\leq 1$, $\omega_{c}$ is the lower boundary cut frequency of passband and *n* is the order of the filter.

(13) $${\left|H\left(\omega \right)\right|}^{2}=\frac{1}{1+\varepsilon^{2}{\left(\frac{\omega }{\omega_{c}}\right)}^{2n}}$$

Considering the trade-off between computational complexity and functionality, we exploit the two-order Butterworth transfer function for filtering, which is denoted by *h*; the original CSI data matrix is *H*; and the filtered matrix is $\bar{H}$; then $\bar{H}=hH$.

### 4.5. Time Delay Filtering Based on the Time Domain

**Figure 6:**
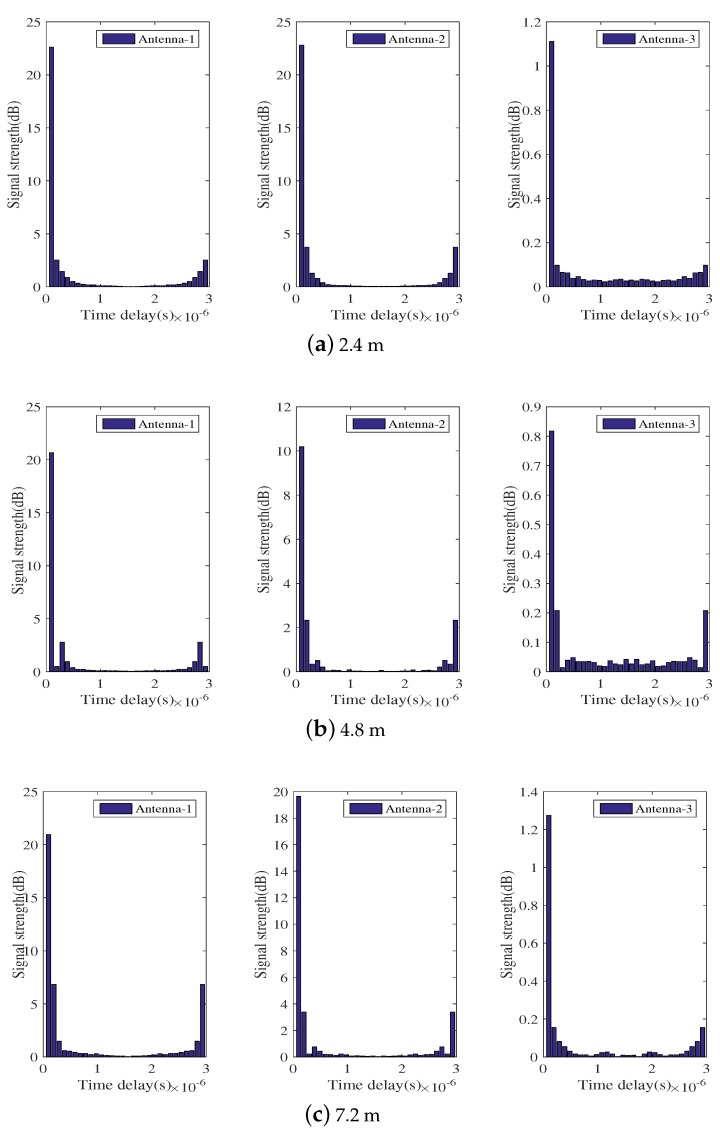
Time domain diagram of the indoor laboratory at different distances after filtering the time delay.

Figure 4 demonstrates that there is a time delay in the CIR graph. At the lowest time, the energy of the LOS path is not the theoretical energy, but the energy value is very small. In order to obtain effective LOS energy, we need to filter the time delay of the time domain CIR graph. Through many experiments, it was observed that the time delay must be in front of a few bars in the CIR energy graph; however, in different situations, the time span is different, and there are always less than five units of the time delay range. According to the CIR graphs, we can see that the span of time delay between indoors and outdoors is less than five time units, and the time delay can be effectively filtered by detecting the first six bars in the CIR graph. Figure 6 depicts the time domain CIR filter after the time delay.

### 4.6. Ranging Model Building

The channel attenuation indicates that the signal strength gradually decreases as the transmission distance increases, which is the main basis for obtaining the signal transmission distance based on CSI. In the transmission system using the 802.11n protocol, the signal is transmitted by multiple subcarriers by leveraging the frequency diversity simultaneously. In general, the wireless signal has its frequency selectively fading, and 30 groups of CSI values are combined at the receiver to combat such multiple frequency subcarriers. The fusion of different subcarrier data can better reduce the error caused by random factors and obtain the true state of the channel. Given a packet with 30 groups of subcarriers, the FILA system [33] proposes the concept of effective CSI, which exploits the frequency diversity to compensate the small-scale fading effect.

(14) $$\begin{array}{c} {CSI}_{eff}=\frac{1}{K}\sum_{k=1}^{K}\frac{f_{k}}{f_{0}}\times {\left|A\right|}_{k}\;,\;k\in \left(-15,15\right) \end{array}$$

where $f_{0}$ is the central frequency, $f_{k}$ is the frequency of the *k*-th subcarrier and ${\left|A\right|}_{k}$ is the amplitude of the *k*-th subcarrier CSI. The propagation distance *d* is then calculated as [30]:

(15) $$\begin{array}{c} d=\frac{1}{4\pi }{\left[{\left(\frac{c}{f_{0}\times |{CSI}_{eff}|}\right)}^{2}\times \sigma \right]}^{\frac{1}{n}} \end{array}$$

where *c* is the velocity of the transmitted wave, σ denotes all other hardware factors (e.g., transmitted power, antenna gains) and *n* is the attenuation factor. The parameters σ and *n* are retrained for each indoor scenario.

### 4.7. Ranging Model Calibration

Due to the complex and changeable geographic layout of indoor space, many obstacles and many sources of interference, the attenuation factor in the ranging model under different environments usually has a great difference.

**Figure 7:**
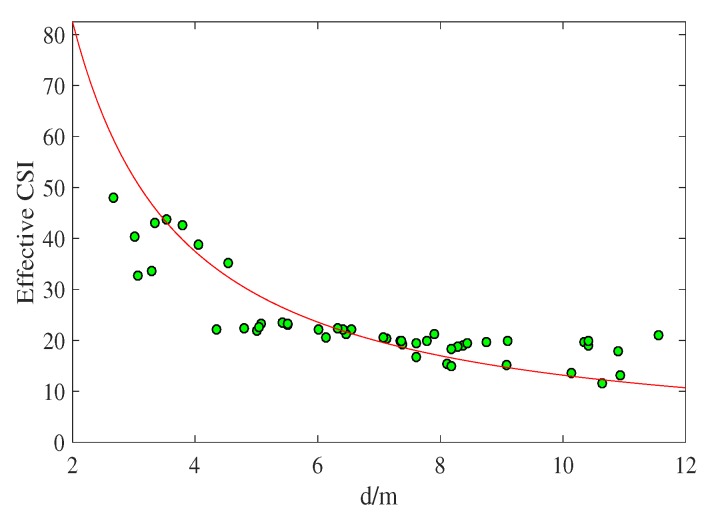
Path loss exponent calculation: *n* = 2.28.

In this paper, we calibrate parameters σ and *n* separately according to the area in which the experiment is conducted. For this experiment, we first use the true distances and the corresponding effective CSI values between special APs and ordinary APs to estimate the value of σ and *n* under the current propagation path. Next, we obtain two metrics by using a large number of measured data processed by the least mean square (LMS) method. An important aspect that needs to be identified before localization experiments is whether the CSI value can establish a relationship with the distance. Figure 7 plots the effective CSI values as a function of distance and illustrates that our ranging model properly fits the relationship between ${CSI}_{eff}$ and *d*. LMS applied to the CSI versus distance data gives that n=2.28. We use this value of *n* to evaluate the SBL-CSI technique.

### 4.8. Algorithm Execution Process

This section combines the above two sections to give the algorithm of SBL-CSI, as shown in Algorithm 1. Firstly, the SBL algorithm is applied to get the location table of each region in 2D space. The perpendicular bisectors among special APs divide the boundary space *S* into three types. Secondly, we use the CSI measurement method to get the location sequence of ordinary AP and then calculate the Kendall’s Tau between the ordinary AP location sequence and the location sequences in table; according to the optimized correlation to obtain the maximum value of $\tau_{max}$. Finally, the centroid of the region corresponding to the Kendall coefficient is the estimated position of the ordinary AP.
**Algorithm 1****Input:** (1) The coordinates of *N* special APs:   $\left\{\left(ax_{i},ay_{i}\right)|i=0\to \left(N-1\right)\right\}$(2) The boundary of localization space *S*;**Output:** Location sequence of all divided regions constitutes location sequence table;1:Use CSI measurement to get effective CSI value between ordinary AP and all special APs;2:**for** each i∈[0,n−1] **do**3:    Collect CSI data between ordinary AP and all special APs;4:    Denoise CSI data by using Butterworth filtering to filter out the high frequency noise;5:    Filter out time delay based on the time-domain LOS path identification method;6:**end for**7:Estimate the distance and calculate the location sequence of ordinary AP;8:Calculate the Kendall coefficient $\tau^{\prime }$ between the ordinary AP location sequence and the sequence in the location sequence table by using Equation (6);9:**for** each i∈[0,n−1] **do**  $\tau_{max}^{\prime }$ = $\tau_{0}^{\prime }$;  $\tau_{max}^{\prime }$ = $\max(\tau_{max}^{\prime },\tau_{i}^{\prime })$;10:**end for**  **return** $\tau_{max}^{\prime }$;11:Choose the centroid represented by the location sequence that has the $\tau_{max}^{\prime }$ with the ordinary AP as its location estimate.

## 5. Evaluations

In this section, we present the implementation and experimental evaluation of SBL-CSI. First, we describe the construction of the experimental scenario, and then, we compare and analyze the positioning results of SBL-RSSI and SBL-CSI in the experimental scene. Subsequently, by using the experimental results, we introduce the criterion to measure the positioning effect of different localization algorithms. Finally, we evaluate the influence of the number of special APs on the positioning error of the two localization algorithms.

### 5.1. Experimental Setup

**Figure 8:**
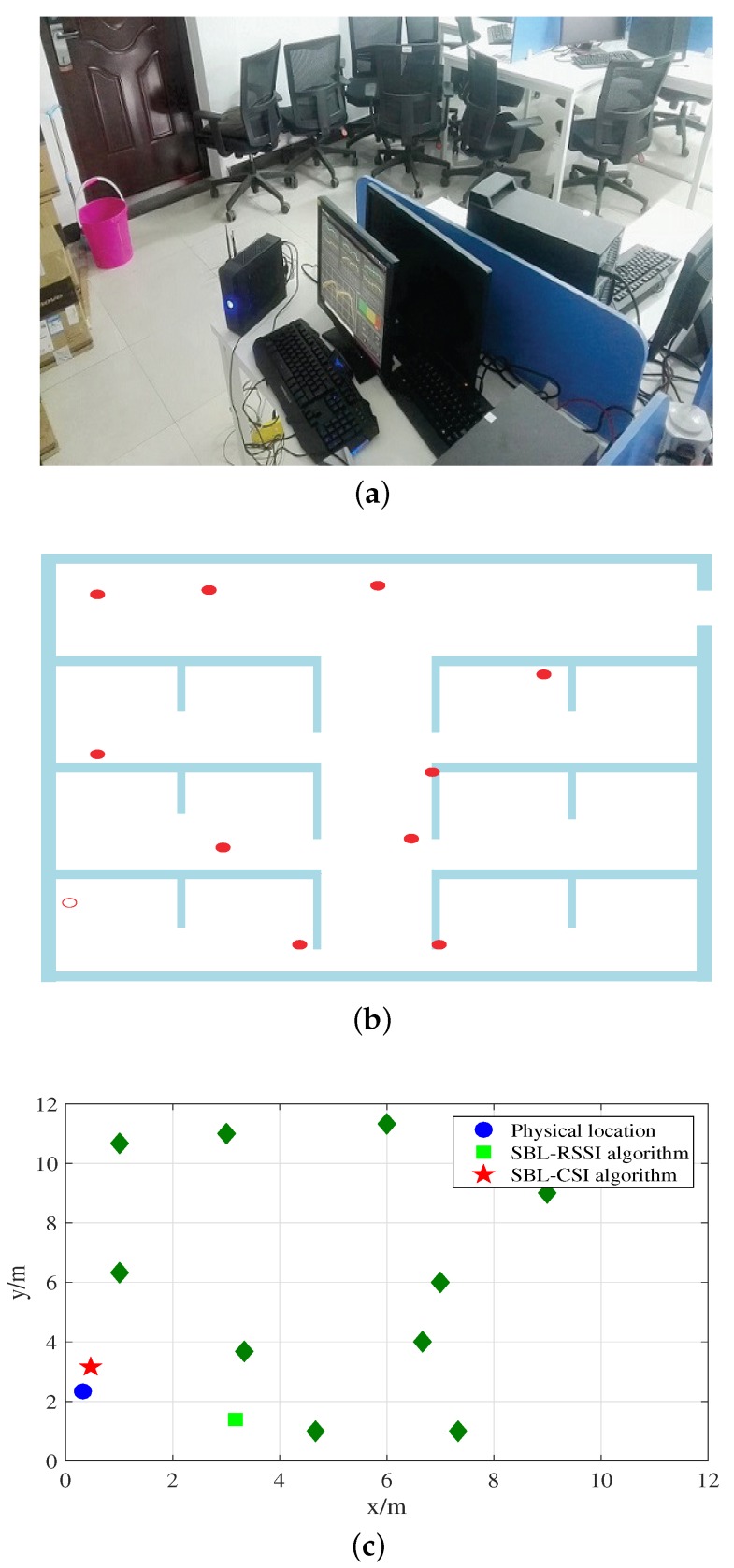
(**a**) Diagram of the experimental scene. (**b**) Diagram of the experimental area. (**c**) Diagram of the ordinary APs and the result.

Based on the concrete working principle of the improved SBL algorithm described above, we deploy three or more special APs to collect CSI information for verifying the validity of the algorithm. Figure 8a shows the experimental scene where we place APs in a square plane of 12 m × 12 m. To compare the impact of the number of special APs on the positioning results, the number of special APs is gradually increased from 3–9. In our experiment, all of the special APs are within each other’s communication range and also within the wireless range of normal APs. We conducted this experiment on a weekday when there were students seating or walking, which demonstrated the robustness of our system to the temporal dynamics of the environment.

As shown in Figure 8b, the solid points represent a special AP, which can parse CSI, while hollow points symbolize ordinary APs whose coordinates need to be estimated. Figure 8c shows the positioning results of 10 special APs to estimate an ordinary AP with two algorithms. Compared to the SBL-RSSI localization algorithm, the SBL-CSI algorithm is more accurate for position estimation. This is because our experiment environment has obstructions blocking the signal transmission, while the SBL-RSSI model requires there to be no obstructions.

### 5.2. Comparison and Analyses

**Figure 9:**
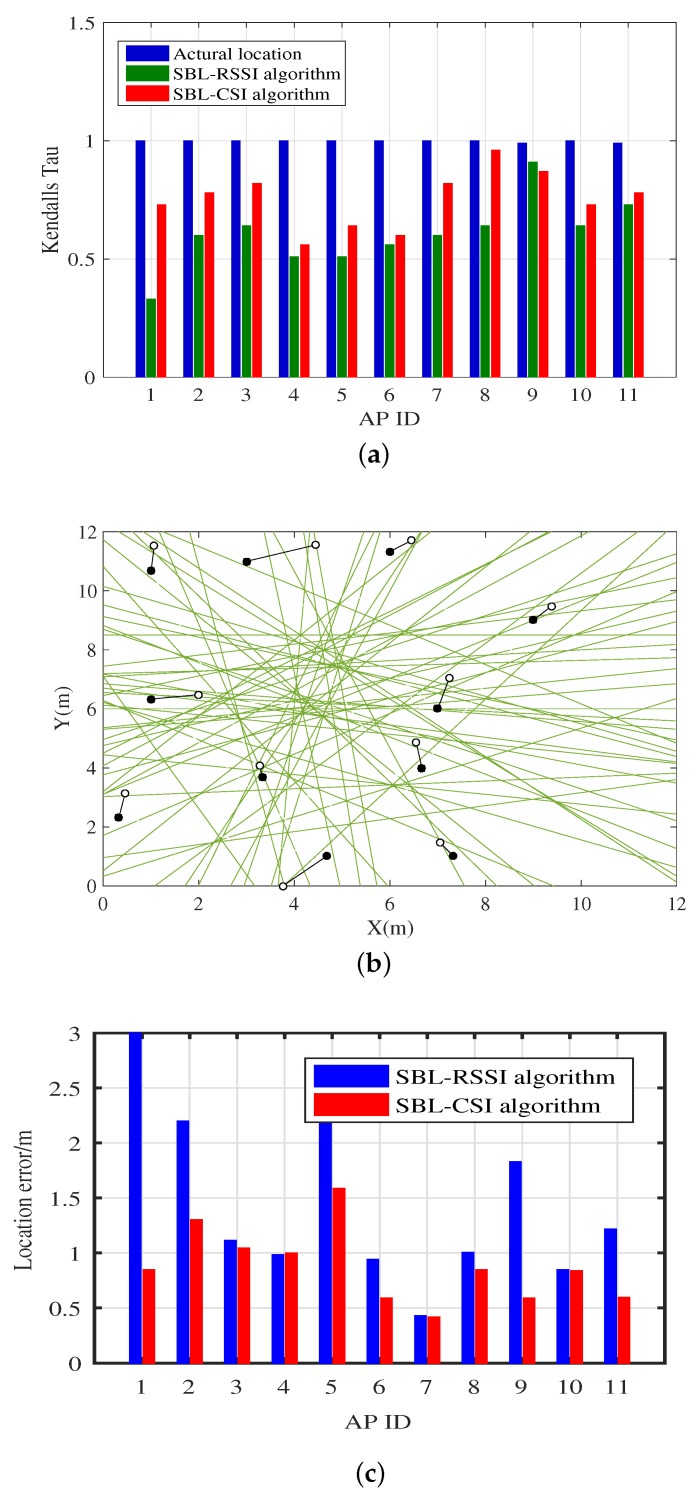
(**a**) Comparison of Kendall’s Tau among the true location, SBL-RSSI and SBL-CSI; (**b**) evaluation of SBL-CSI algorithm; (**c**) comparison of the positioning error of the SBL-CSI and SBL-RSSI algorithms.

The positioning results and positioning error are shown in Figure 9. The impact of multipath fading effects on RSSI measurement can result in erroneous ranking of ordinary AP location sequences, further leading to erroneous estimation of their coordinates. The value of τ reflects the correlation between two sequences; the closer this value is to one, the smaller the difference between two sequences is, and the location sequence mapping to the centroid of region is closer to the located point. We can see from Figure 9a that the value of the SBL-CSI algorithm is closer to one than that of the SBL-RSSI algorithm, that is the location sequence of the SBL-CSI algorithm is hardly uncorrupted. Figure 9b illustrates the difference between the physical location of the AP (solid point) and the estimated location of the SBL-CSI algorithm (hollow point), and it also shows the arrangement of perpendicular bisectors between all special APs. Figure 9c shows the positioning error of the two algorithms; for each AP’s positioning error, the SBL-RSSI algorithm outperforms SBL-CSI algorithm.

### 5.3. Performance Study and Evaluation

In the case of the positioning effect evaluation, location error is a profound reference, and location error generally refers to the Euclidean distance between the estimated location and the physical location. The location error is described as:

(16) $$\begin{array}{c} L_{e}=\sqrt{{\left(x_{e}-x_{r}\right)}^{2}+{\left(y_{e}-y_{r}\right)}^{2}} \end{array}$$

where the estimated location is presented as $\left(x_{e},y_{e}\right)$ and $\left(x_{r},y_{r}\right)$ stands for the physical location.

**Figure 10:**
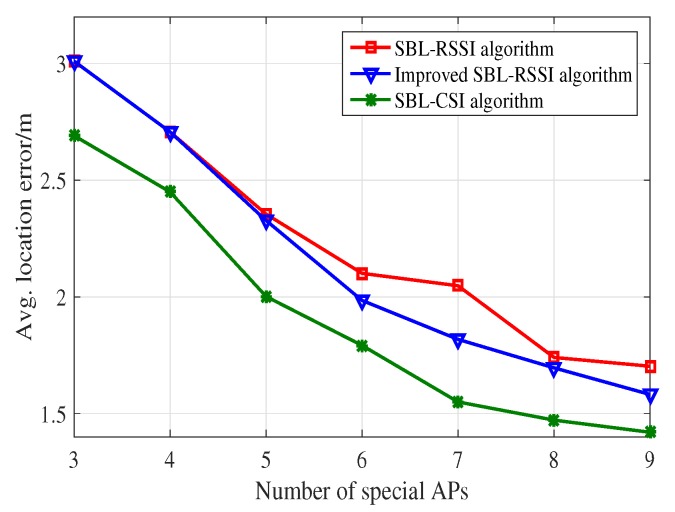
Average location error in the SBL-RSSI algorithm, the SBL-RSSI algorithm with improved Kendall’s Tau and the SBL-CSI algorithm.

Since different numbers of APs have a great influence on the positioning results, we evaluate the performance of the algorithms fairly and effectively by using the average location error. To assess the effectiveness of the SBL-CSI localization approach, in the following, we compare the performance of our localization system with the counterpart SBL-RSSI method. The experiment is conducted in the laboratory where we place three or more special APs. In our experiment, there are many desks and chairs, even walking students. Figure 10 illustrates the average location error at 11 coordinate points. In such a large laboratory with many obstacles causing the multipath effect, the SBL-CSI algorithm has better performance, which indicates that the fine granularity of CSI is beneficial to improve the accuracy of the indoor localization approach.

The set of experiment aims to study the effect of the number of special APs on the system performance. Figure 10 shows that, as the number of APs increases, the average location error of the SBL-RSSI algorithm, the refined SBL-RSSI algorithm with the improved Kendall’s Tau and the SBL-CSI algorithm decreases, and the localization result is more accurate. Comparing the SBL-RSSI algorithm with the refined SBL-RSSI algorithm, we can see that the Kendall coefficient has an influence on the average location error. The reason is that the length of the distance ranks will increase when the number of special APs increases, and the average location error will decrease. The trend of the numerical value reflects that the average location error of the SBL-CSI algorithm is obviously lower than that of the other two algorithms. For example, with seven APs, SBL-CSI obtains a 24.31% lower average location error compared with SBL-RSSI; additionally, the average location error for SBL-CSI is about 9.71% less than the refined SBL-RSSI. The experimental results show that the performance of the WLAN access point location can be improved by increasing the number of special APs, and compared with localization systems based on RSSI, localization schemes based on CSI have a higher localization accuracy.

## 6. Conclusions

Indoor localization is one of the most promising technologies that can be widely applied to many fields, where RSSI-based schemes, for indoor localization, have drawn much attention. RSSI is easily affected by multipath fading. In this paper, we introduce SBL-CSI, a novel scheme based on fine-grained information, which uses channel state information for the accuracy of AP localization. We have conducted extensive experiments in the laboratory to prove the effectiveness of CSI in complex environments. The experimental results show that the accuracy of average location error can be significantly reduced by using CSI.
